# A Real-Time All-Atom Structural Search Engine for Proteins

**DOI:** 10.1371/journal.pcbi.1003750

**Published:** 2014-07-31

**Authors:** Gabriel Gonzalez, Brett Hannigan, William F. DeGrado

**Affiliations:** Cardiovascular Research Institute, University of California, San Francisco, San Francisco, California, United States of America; UCSD, United States of America

## Abstract

Protein designers use a wide variety of software tools for *de novo* design, yet their repertoire still lacks a fast and interactive all-atom search engine. To solve this, we have built the Suns program: a real-time, atomic search engine integrated into the PyMOL molecular visualization system. Users build atomic-level structural search queries within PyMOL and receive a stream of search results aligned to their query within a few seconds. This instant feedback cycle enables a new “designability”-inspired approach to protein design where the designer searches for and interactively incorporates native-like fragments from proven protein structures. We demonstrate the use of Suns to interactively build protein motifs, tertiary interactions, and to identify scaffolds compatible with hot-spot residues. The official web site and installer are located at http://www.degradolab.org/suns/ and the source code is hosted at https://github.com/godotgildor/Suns (PyMOL plugin, BSD license), https://github.com/Gabriel439/suns-cmd (command line client, BSD license), and https://github.com/Gabriel439/suns-search (search engine server, GPLv2 license).

This is a *PLOS Computational Biology* Software Article

## Introduction

Protein structural bioinformatics rapidly approaches a big data crisis as the last decade has witnessed a dramatic increase in protein structure depositions. In 1993 researchers had just over 28,000 searchable structures at their disposal in the Protein Data Bank (PDB), while today we have over 101,000. This rapid structural expansion could inform protein design, structure determination, and structure prediction by providing numerous examples of native-like structural interactions in exquisite detail, but researchers lack high-powered computational tools to intelligently search and explore large structural data sets in detail.

One of the first popular protein structural search tools developed for this purpose was Dali by Holm and Sander [1]. Dali uses distance maps formed by calculating pairwise α-carbon distances to form a two-dimensional representation of a three-dimensional protein. Regions of similarity between two distance maps correspond to similar substructures in their respective proteins. Holm and Sander used Dali to create the Families of Structurally Similar Proteins (FSSP) database [2], which aligns substructures across entries in the Protein Data Bank (PDB) to form families and subfamilies of common folds. Researchers commonly use Dali to compare protein folds and infer homology [3]–[5] and similar algorithms specialized to structural comparison and similarity detection include combinatorial extension (CE) [6], sequential structure alignment program (SSAP) [7], and TM-align [8].

The more recent MaDCaT search program [9] also uses α-carbon distance maps to search for similar protein backbone arrangements. However, where Dali uses a heuristic approach to detect structural similarity, MaDCaT takes a query backbone structure or motif and finds globally optimal structural matches within an entire structural database. This approach makes MaDCaT ideal for finding the best matches to frequently occurring motifs. These “designable” motifs promise to be excellent design scaffolds, and MaDCaT applied this approach to design a viral-like protein coat for carbon nanotubes from designable interactions [10].

Both Dali and MaDCaT return results after several minutes of searching. For greater speed, Shyu et. al. developed ProteinDBS [11] in order to provide the first real-time protein backbone search. They use image processing techniques to extract a set of features from α-carbon distance maps and organize their structural database into a tree, allowing quick traversal and parallelism during searches. These optimizations allow them to return search results nearly instantly, but they limit themselves to searching for backbone α-carbons.

We required an all-atom search engine to guide the protein design process, so that we could search for proteins with similar active sites or binding motifs, explore protein scaffolds that can host a specific motif, and discover atomic-scale supporting interactions.

The state of the art for all-atom search is Erebus [12], which permits all-atom rigid substructure searches and has been used to identify ligand interaction motifs for drugs and metal ions. However, Erebus is not ideal for design purposes where an interactive search process is desired. Several bottle-necks in the Erebus search workflow impede a fluid design process, including time-consuming assembly of search queries, long search delays, and a non-programmatic web interface for retrieving results.

A truly interactive search tool must remove every single one of these bottlenecks to bring the feedback loop down from minutes to seconds and permit users to rapidly explore multiple design alternatives iteratively in atomic detail. Improved speed and faster feedback lets researchers ask more sophisticated questions, explore structures more intelligently, and use limited collaboration time more efficiently.

The Suns protein search engine makes it easy to search and browse a database of protein structures at the atomic level. To our knowledge, Suns is the first real-time all-atom structural search engine and is also the first to integrate seamlessly into the popular molecular visualization program PyMOL [13], so that researchers can easily click on motifs of interest, click search, and view aligned results within a fraction of a second. We expect Suns to inform and guide protein design, modeling, and structure determination by lowering the entry barrier to structural search so that it becomes a staple of every structural biologist's toolbox rather than a tool limited to programmers.

## Design and Implementation

### Overview

Protein substructure search is a special case of sub-graph isomorphism, which is NP-complete in the general case [14]. Therefore, substructure search tools will usually reduce this combinatorial complexity by either restricting permitted search queries or taking advantage of properties specific to graphs of protein atoms.

Our structural search engine greatly resembles a web search engine, even though these two types of engines index different types of data: web search engines commonly index linear text strings whereas our search engine indexes three-dimensional protein structures. Despite these differences, we still borrow many principles from web search engines [15] to improve search speed:

1. Divide structures into structural “pages” (3-D volumes) analogous to web pages
2. Divide these “pages” into structural “words” (chemical motifs) analogous to textual words
3. Create a forward index that matches sets of structural words to structural pages
4. Perform slower and more accurate filters after the fast forward index lookup
5. Return only as many results as requested to avoid unnecessary computation

**Figure 1:**
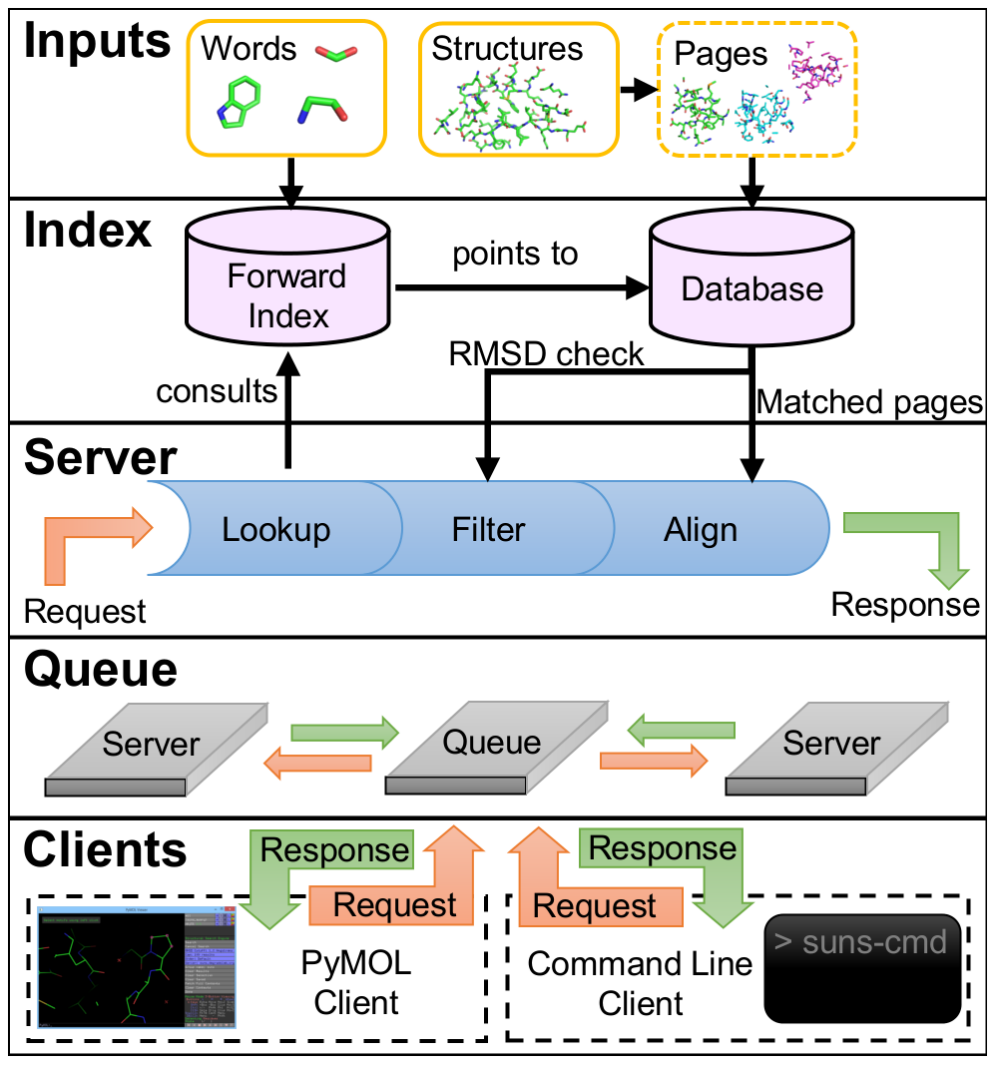
Overview of Suns algorithm and architecture. (Inputs) The search index is built from two inputs: a set of words to recognize and a set of protein structures to search subdivided into pages. (Index) The two underlying data structures are a forward index that translates words to matching pages and a database of every page which translates matched words to atoms within each page. (Server) Each request to the server is broken into three steps: consult the forward index to find potentially matching pages, filter matching pages by RMSD to the query, and aligning successful matches to the query. (Queue) A message queue forwards requests from clients to servers, and forwards responses from servers to clients. (Clients) Suns provides two client interfaces: a PyMOL search plugin and the suns-cmd command line interface.

The search engine is organized using a client-server architecture (Figure 1). The search engine and storage are written in Haskell, taking PDB files to index as input and writing a custom binary format to disk. We provide two clients, one of which is a PyMOL search plugin written in Python and the other of which is a command line program written in Haskell. The search engine communicates using JSON queries through a custom request/response format mediated by a RabbitMQ-based message queue.

### Forward index

Web search engines derive much of their speed by preprocessing the data set using a forward index that matches words to web pages [15]. The search engine can then tokenize each query into words and consult the forward index to rapidly return all pages that contain every word in the user's search query. Protein search engines can copy this trick, but they must first decide what volume size corresponds to a “page” and what chemical motifs correspond to “words”.

Two opposing considerations constrain the choice of page and word size. The forward index resolves pages solely by their word counts, so larger words and smaller pages lead to more unique word counts per page and improves the selectivity of the forward index. However, users prefer the exact opposite: smaller words and larger page sizes increase the power and flexibility of user search queries. Therefore, optimizing a structural search engine requires balancing user needs against the efficiency of the forward index.

**Figure 2:**
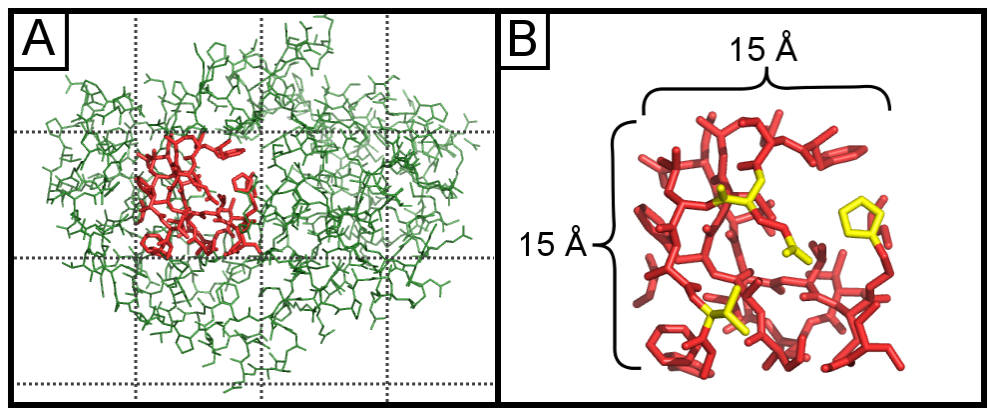
Subdivision of protein structures. (A) An interior page highlighted in red from a protein of unknown function (PDB ID  =  2FSQ), illustrating the maximum scale of search queries. (B) Example words (chemical motifs) within the same page highlighted in yellow. Pages are 15 Å×15 Å×15 Å cubes.

We select a compromise suitable for atomic-level search queries: we restrict structural pages to cubes 15 Å wide and we define structural words to be connected chemical substructures ranging from 2 atoms (a hydroxyl) to 9 atoms (an indole ring) (Figure 2). Our choice of page size assumes that larger structural patterns of interest can be reduced to a network of bridging local interactions below the 15 Å length scale. Similarly, our choice of word size assumes that users will accept a modest restriction on search queries to groups of chemical motifs instead of groups of atoms. Like web search engines, we permit searches for multiple disconnected words, allowing users to assemble complex queries from these simple chemical building blocks.

Protein structures are divided into non-overlapping pages, meaning that search queries will not return results spanning multiple pages. While this necessarily means that queries will miss some potential matches, we have found that in practice most queries still find more matches than requested by the user. Moreover, by simplifying the database in this way, we avoid having to add an additional post-processing step to eliminate duplicate results.

### Structural words

We specify structural words using PDB files, which contain the specific residue and atom types to match. For example, one structural word consists of a single PDB file containing the Cα-Cβ-Cγ that links the phenyl group of phenylalanine to its backbone atoms. When users search for the three carbon atoms in phenylalanine's linker, their searches will not match tyrosine's linker, nor will they match three connected ring carbons within a phenylalanine. This allows the search index to optionally resolve motifs that are otherwise chemically identical [16]. The index identifies words solely by their connectivity graph and not their geometric similarity - thus cis/trans isomers cannot be resolved at this level of representation.

Structural words may also match more than one protein element, and in those cases we use multiple PDB files to specify the structural word: one PDB file per matching chemical motif. For example, one motif we index is a carboxylate, specified using two PDB files we created: one for glutamate's carboxylate and another for aspartate's carboxylate. User search queries for carboxylates will match either of these two groups.

The choice of structural words is customizable and for our public-facing server we select a default set of 28 substructures appropriate for general-purpose searches (Table S1). The most important searchable substructure matches the four backbone atoms for any protein residue, which permits geometrically precise backbone searches that specify the positions of all backbone atoms and their torsion angles. We partition flexible residues such as lysine and methionine into two separate words, and also isolate important chemical moieties into their own words, such as imidazole and guanidinium groups. Some chemical moieties are shared between residues, such as the hydroxyl group, which matches serine, threonine, and tyrosine. However, every residue except glycine possesses at least one unique structural word so that users can restrict searches to a specific residue.

### Database

Our forward index is formally a *record level* inverted index that converts sets of words to matching pages. We supplement the forward index with a custom in-memory database that stores two pieces of information necessary to complete the search. First, the database stores correspondences between words in the forward index and atoms in each structural page. Second, the database also keeps compact representations of every structural page suitable for returning as search results

When the forward index produces a matched page, the database remembers which atoms in that page correspond to the words advertised in the forward index. Sometimes the page contains more instances of a given word than the user required, such as when the user searches for two peptide bonds, and the page contains five. The page must try out every valid permutation of words that match the user's query, and the forward index minimizes the number of permutations by prioritizing pages that closely match the minimum required word count.

### Alignment and RMSD

Suns uses the Kabsch algorithm [17] to rapidly align each permutation to the user's search query. The Kabsch algorithm requires an exact atom-for-atom correspondence between the user's search query and a candidate motif, and Suns compiles this correspondence from precomputed atomic correspondences for each stored motif in the custom database. After alignment, the search engine only returns search results that match the search query within a specified root-mean-square deviation (RMSD) cutoff.

For each result below the RMSD cutoff, Suns aligns the matching page to the search query and returns the page as the search result. If a page contains multiple matches Suns aligns each match separately and returns them as separate results. This superimposes every search result and context on the original query for ease of visual comparison and downstream post-processing.

### Streaming results

The user may dial in the stringency of desired matches by tuning the RMSD cutoff. The search engine will immediately stream any result within this cutoff, which allows the user to begin visualizing results before the search has completed, improving interactivity.

Additionally, the search protocol requires the user to specify the number of desired results up front. While the user may request an unlimited number of results in theory, in practice the search clients default to 100 search results, similar to how a web search engine will default to 10 search results. This allows the search engine to stop processing the request after supplying the specified number of results, which reduces server load. The search engine may also optionally specify a search timeout to further reduce server load for users that request a large number of search results.

### Data set

The public search engine uses the most stringent precompiled dataset from the PISCES [18] non-redundant protein structure datasets, selecting a 20% sequence identity, 1.6 Å resolution, and 0.25 R-factor cutoff, which currently comprises 2058 chains. The search engine's available memory limits how many structures it can index, and our stress tests on the largest precompiled PISCES data set (90% identity, 3.0 Å, 1.0 R-factor cutoff, 24,218 chains) required 89 GB of memory or an average of 1 GB of memory per 272 protein chains.

During informal testing, we found the larger data set was unable to discover novel motifs absent from the more stringent database. Consequently, we selected the smaller data set in order to maximize the diversity of search results (by reducing sequence identity) and minimize memory consumption. One thing to note is that as the PISCES dataset is comprised of single chains, our default database does not index motifs from inter-chain interactions. Therefore, users searching for protein-protein interactions may wish to customize the search engine to index PDB files specially curated for interaction motifs.

## Results

### Building motifs: Incremental salt bridge assembly

**Figure 3:**
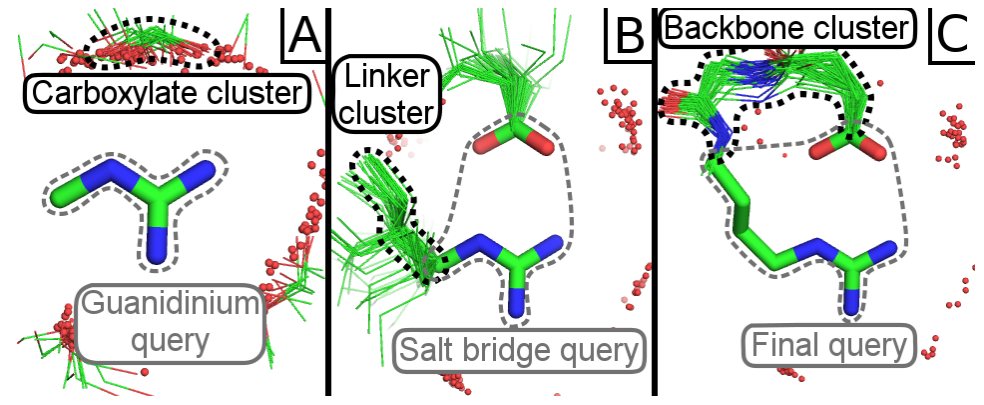
Incremental assembly of a motif. (A) An initial search for a guanidinium fragment reveals a cluster of nearby carboxylates. (B) Refining the search with one carboxylate from the results reveals a specific linker preference for both the aspartate and arginine involved in the salt bridge. (C) Adding the most common linker for arginine and repeating the search reveals that this salt bridge is part of an N-terminal capping motif. Search queries are represented as thick sticks and search results are shown as thin lines. Grey dashed lines highlight search queries and black dashed lines highlight clusters in the search results, which are filtered to show the specific residue fragments of interest and neighboring water molecules within 3.0 Å as red spheres. Search parameters and fragments listed in Table S2.

Suns lets users explore the “designable” space of protein motifs, defined as naturally reoccurring substructures, by expanding on small initial fragments, such as building a helix N-terminal capping motif beginning from a single guanidinium group. One might begin by searching on the guanidinium fragment from an arginine, which recruits a cluster of nearby carboxylates forming a salt bridge with the arginine (Figure 3A). Adding one of these carboxylates to the search query refines the motif further, revealing a preferred rotamer for the arginine when interacting with a carboxylic acid (Figure 3B), and adding a preferred rotamer to the search query crystallizes a complete N-terminal capping motif (Figure 3C).

The large number of close geometric matches to the final search query suggests that this is a highly “designable” motif. Incremental searching allows users to rapidly explore and prototype designable native-like interactions like these with very little prior knowledge in protein folding or biophysics. Moreover, a user can discover the motif by gradually refining a specification rather than specifying all the necessary interactions up front. This benefits people who may not even know what designable interactions look like and simply wish to explore what options they have available. We recorded an example of this iterative search and discover process as Video S1.

The salt bridge we built this way also matches one of many newly discovered salt bridges by Donald et. al (Figure 8 of [19]). However, we identified this without requiring a curated database of salt bridges and without using a specialized algorithm built to detect electrostatic pairs. We also obtain detailed information from the superimposition of results, which allows us to visualize the structural variability of this salt bridge motif on a per-atom basis.

### Assembling larger fragments: A designable beta sheet built from 9 isolated residues

Users can build tertiary interactions for proteins as well. To demonstrate this, we search for a valine from glucose binding protein and grow that into three small β strands with three residues per strand.

**Figure 4:**
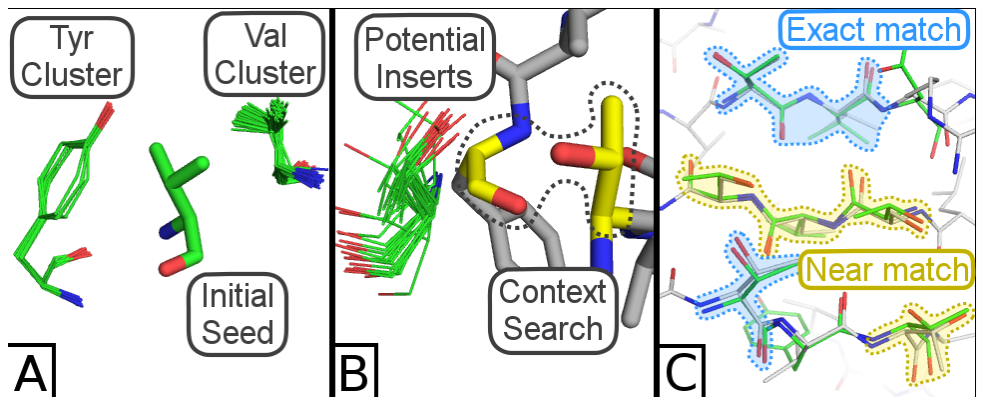
Building a tertiary interaction. (A) Three strands are seeded by searching on a valine, which reveals two nearby clusters of valine and tyrosine. (B) Strands are extended one residue in each direction by searching for pairs of residues (colored yellow) in the context of an insertion site, yielding clusters of potential inserts (colored green). (C) The final backbone fragment (green) is fed to MadCaT, which identifies multiple compatible scaffolds. One such scaffold (PDB ID = 1E54, colored light grey) possesses many exact residue/rotamer matches to the assembled fragment (blue highlights) and many close matches (yellow highlights) that differ by a related residue (threonine to serine or valine to isoleucine) or by varying the rotamer.

Beginning from an interior valine from glucose-binding protein, we seed the two adjacent β strands with highly populated residue clusters on each side corresponding to a valine and tyrosine (Figure 4A). To grow the three β strands in both directions, we search for pairs of residues at a time to identify new clusters of residues within the search results that we can insert into the sheet (Figure 4B). The PyMOL search client permits a qualitative inspection of residue preference at selected positions by cycling through visualizing each residue type. This process not only provides a rough measure of residue preference, but also reveals rotameric preference, the kind of detailed information that a sequence logo would not reveal.

We repeat this process of iteratively searching for pairs of residues at a time and incorporating clusters from the search results until we assemble a native-like fragment of a sheet where almost every residue originates from a unique protein structure (two disconnected threonines were inadvertently drawn from the same structure). This then provides α-carbon coordinates that we feed into the backbone search engine MaDCaT [10], which finds suitable scaffolds to incorporate this fragment. One MaDCaT search result greatly resembles the β sheet built using Suns (Figure 4C).

### Connecting hot spot residues: Recapitulating a hemagglutinin binding motif

Suns can also be used to find scaffolds compatible with specified residues to provide an alternative implementation of the hotspot residue approach to design [20]. The user can select the hotspot of interest within PyMOL, search, and find several protein structures in the PDB that position the given hot spot residues in the specified geometry.

**Figure 5:**
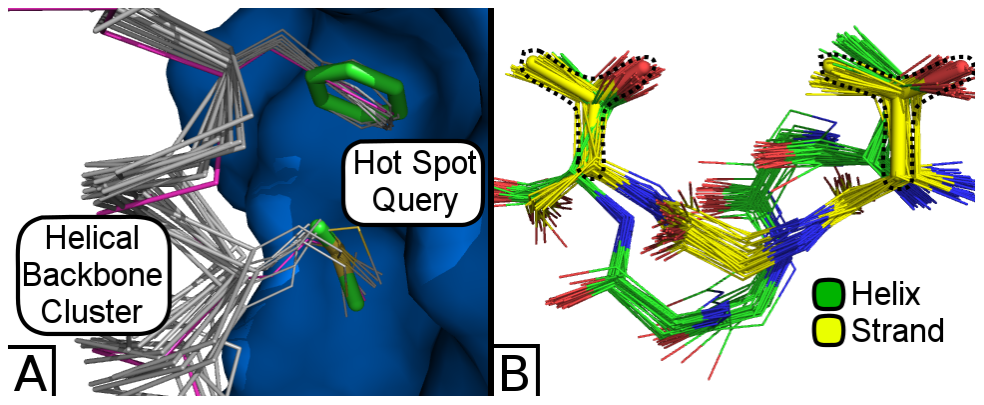
Finding backbones compatible with hot spot residues. (A) A Suns search at 0.7 Å RMSD cutoff for two hotspot residues previously identified by RosettaDock [22] for a hemagglutinin binder [20]. The majority of search results are helices that closely match the final designed protein. The search query is shown in thick green sticks, the search result matches are shown as grey α-carbon traces, and the designed hemagglutinin binder is shown as a purple α-carbon trace against a blue hemagglutinin surface. (B) Searching for two threonine side chains at 0.6 Å RMSD cutoff reveals two backbone clusters that can connect them, one corresponding to an α helix (green) and the other corresponding to a β sheet (yellow). Black dashed lines surround the original search query, which is represented as thick yellow sticks.

For example, Suns recapitulates the local backbone of a designed hemagglutinin binder [20]. Figure 5A illustrates how searching for fragments of the original hotspot residues reveals a prominent cluster of α helices matching the designed protein structure, indicating that the secondary structure of the interface could have been predicted solely from designability.

Not every hotspot search will return a single solution for the backbone. Sometimes searching for disembodied residues will reveal multiple distinct ways to thread the backbone between them (Figure 5B).

### Metal binding sites: Searching for a subset of the EF-hand motif

Suns can complete metal binding sites when provided with a small subset of the original motif. We begin from an EF-hand from calmodulin (PDB ID  =  1CLL) and search for only two aspartate side-chains from the motif, each of which coordinates the calcium ion once. Note: this search does not include the metal ion.

**Figure 6:**
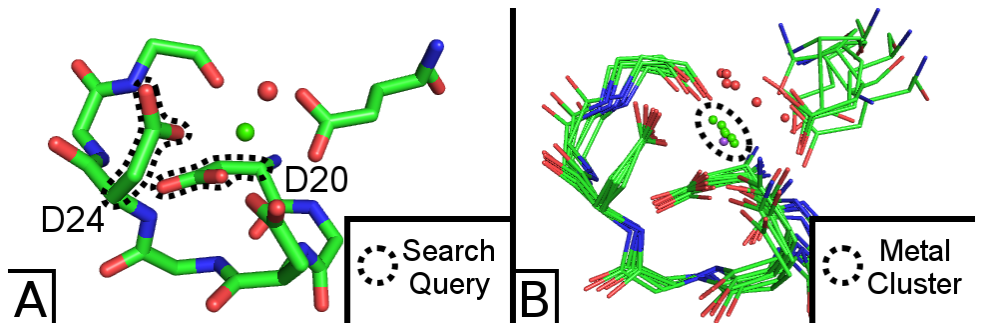
Searching for calcium binding sites. (A) Two side chains of the EF-hand of calmodulin suffice to find matching motifs. The search query (black dashes) consists exclusively of two aspartate side chains (D20 and D24) and does not include the calcium ligand. (B) Searching for these two side chains at 0.7 Å resolution returns seven results, all of which are EF-hand motifs. Six of these motifs coordinate a matching calcium ion (green sphere), and the seventh motif coordinates a sodium ion (purple sphere).

Searching for these two aspartate side chains at an RMSD cutoff of 0.7 Å returns seven search results (Figure 6). Each one of these results is an EF-hand motif that aligns closely to the original search query. Even though the search query did not include the metal ion, every result coordinates a calcium ion at the same location as calmodulin, with the exception of one result which coordinates a sodium ion.

### Benchmarks: Latency

**Figure 7:**
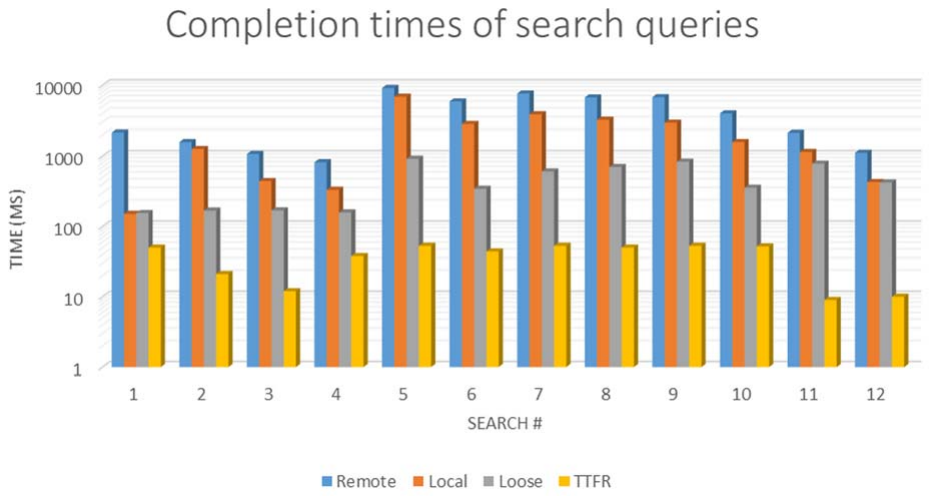
Latency benchmarks. We benchmark completion times of searches for: (Remote) low (<0.6 Å) RMSD cutoff queries against our public server, (Local) low RMSD cutoff queries against a local server, and (Loose) queries with a default RMSD cutoff (1.0 Å) against a local server. We also measure the time to first result (TTFR) under the same conditions as (Loose) queries. Each query corresponds to a specific search illustrated in the *Results* section and the query PDB files are included as part of the benchmark suite of suns-cmd (Software S2).

Suns is optimized to minimize latency to support an interactive workflow, so we benchmark the turnaround time for queries under increasingly optimal scenarios. The suns-cmd source code includes a benchmark suite that tests the speed of searches taken from the above example sections. We begin by benchmarking against the public search engine over a network connection, then benchmark successive improvements to query speed by (A) testing against a server deployed on the same machine as the client followed by additionally (B) loosening the RMSD cutoff to 1.0 Å, our default cutoff. For each of these scenarios, we record the total time from the start of the query until the final result is returned, but for an interactive tool like Suns, the amount of time between the start of the query to the first result is also of great interest, so we also record (C) the time-to-first-result (Figure 7). The networked server runs on a single Intel Xeon E5-2420 1.90 GHz core, while the local server runs on a single Intel i5-3230M 2.60 GHz core.

The slowest queries are performed over the network and use stringent RMSD cutoffs (<0.6 Å), yet they still produce 100 results between 1 to 10 seconds. Loosening the RMSD to the default RMSD cutoff of 1.0 Å and improving the network connection to the best-case scenario of a local server improves speed approximately 10-fold, reducing search time to between 100 ms and 1 second. However, Suns often “feels” even faster than that because of its rapid time-to-first-result (TTFR), which ranges from 10 ms to 100 ms, allowing the user to begin browsing aligned results immediately while they continue to concurrently stream into PyMOL.

We do not formally compare Suns latency to Erebus because Erebus does not yet provide a programmatic interface for result submission or retrieval and all results must be downloaded individually by hand. Therefore the overhead of submitting an Erebus query and retrieving search results is minimally on the order of several minutes for tens of results, without including search time. Additionally, Erebus is not optimized for rapid subsequent searches since Erebus exhaustively scans the entire PDB for every search.

### Benchmarks: Throughput

An accurate comparison between Suns and Erebus is difficult due to differing design tradeoffs between the two servers. One of Erebus's strengths is its comprehensiveness: Erebus searches can span the entire protein data bank. In contrast, Suns chooses to search a non-redundant set of structures from the PDB so that the entire database can fit in the server's working memory (currently 96 GB) minimizing performance issues involved in disk I/O. Therefore, we estimate database throughput for both search engines by normalizing query times using the size of the data set that each search engine indexes. For these throughput benchmarks we test against a locally hosted Suns server which indexes a data set of approximately 2,000 models, whereas the self-reported throughput of Erebus [12] is based on a data set of approximately 200,000 models (100-fold larger). Also note that the original Erebus benchmark uses 16 2.10 GHz cores whereas Suns runs on a single 2.60 GHz core for this benchmark.

**Figure 8:**
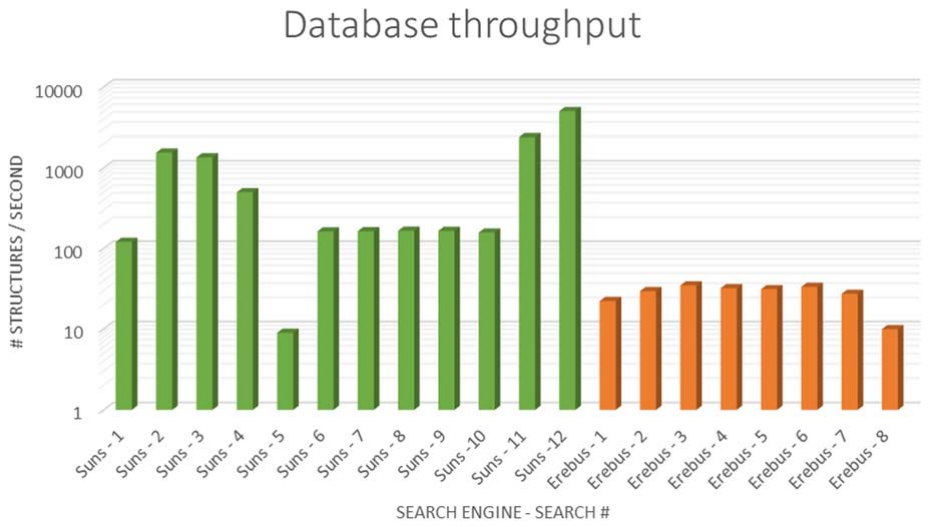
Throughput benchmarks. We compare throughput of search queries for both Suns and Erebus, defined as query time divided by number of models in the data set. Suns throughput is measured against a locally hosted server and the Erebus throughput data is taken from [12]. Detailed query information, including the query size in atoms and the number of matches, is provided in Table S3 and the specific query PDB files are included in the benchmark suite of suns-cmd (Software S2).

For both Suns and Erebus, the worst-case throughput for the example search queries is 9–10 structures/second, meaning that every 10 structures in the database extends the search time by 1 second. However, every other Suns query outperforms Erebus in throughput by one or two orders of magnitude, and many of them process over 1000 structures per second and return hundreds of matches (Figure 8).

Most of these efficiency gains for Suns can be attributed to how well search queries take advantage of the forward index. The best performing search #11 includes a methionine, a residue of low natural abundance, alongside a phenylalanine, so we expect this search to utilize the forward index well and eliminate most pages quickly. In contrast, the worst performing search #5 comprises a highly degenerate query: two backbone motifs. This query performs poorly because it combines two motifs of extremely high abundance, forcing Suns to inspect nearly the entire data set in detail. Normally, high abundance queries also perform well for Suns because they saturate the requested number of results rapidly, but this benchmark explicitly instructs Suns to continue to produce as many results as possible to measure dataset throughput.

### Conclusions

Our primary contribution is an entire graphical design workflow designed from the ground up to allow rapid and interactive exploration of large structural design spaces. We accomplish this by reducing performance and interface bottlenecks in order to encourage users to issue multiple search queries in rapid succession. Consequently, people can easily tailor Suns to their specific needs by composing multiple small searches instead of constructing a single monolithic search query with a large configuration space. Additionally, the first-class support for graphical search and feedback provides greater opportunities to inject human intelligence into the search process.

We initially built Suns to guide the protein design process, but we are releasing it as a general-purpose search engine so that others may reuse it for applications we did not previously anticipate. Drug discovery researchers may benefit from generalizing Suns to index ligand substructures to discover favorable protein-drug interactions. Also, modelers may use Suns as a generalized PROCHECK [21] to independently validate small structural regions that are insufficiently constrained by the data.

Compared to the Erebus atomic substructure search engine, Suns primarily innovates on latency, throughput, search volume, and interactivity, but at the price of data set coverage and restricted queries. Suns originated as a rapid prototyping tool and many of the design tradeoffs reflect an emphasis on performance rather than completeness. However, Suns may be able to scale to cover the entire Protein Data Bank by layering an additional distributed apparatus on top of the search engine. The search algorithm is “embarrassingly parallel” so memory limitations can be circumvented by distributing the search workload across several machines, each of which indexes a subset of the Protein Data Bank that fits within memory. Additionally, the current page partitioning scheme could be modified to include staggered and overlapping pages to guarantee complete motif coverage.

We place a high importance on ease of integration and distribution to encourage other projects to build upon and customize Suns. Suns provides programmatic access through libraries and command line clients for ease of incorporation into derived, automated workflows. Suns is also fully open source and allows users to host their own local search engine to improve performance or tailor the search engine to their needs. Locally deployed search engines will particularly benefit commercial enterprises which cannot afford to transmit sensitive proprietary data outside of their intranet.

Suns can piece together designable fragments such as beta sheets that can in turn be fed to coarse-grained search engines such as ProteinDBS or MaDCaT. Unifying these complementary tools might allow users to seamlessly transition between diverse length scales as designed protein fragments grow in size.

## Availability and Future Directions

The Suns plugin for PyMOL is available at www.degradolab.org/suns, which also includes a tutorial on how to install and use the library (Manual S1). The source code for the client is available separately at https://github.com/godotgildor/Suns under a BSD license (Software S1).

Users can also automate searches using a command line tool, available at https://github.com/Gabriel439/suns-cmd under a BSD license (Software S2). Users who wish to incorporate Suns within an automated workflow should use this client instead.

The source code for the search engine is located at https://github.com/Gabriel439/suns-search under a GPLv2 license (Software S3). Users should report bugs or request new features using the issue tracker at https://github.com/Gabriel439/suns-search/issues or by contacting the Suns mailing list at suns-search@googlegroups.com.

Currently the public search engine only indexes protein structures. We also plan to add support for ligand search queries so that Suns can be used for drug design. While this paper describes a protein-specific application of the search engine, the underlying algorithm can be readily generalized to ligands and other macromolecules.

## Supporting Information

Table S1 **Default motif set.** Default motifs indexed by the public server hosted at suns.degradolab.org. (Motif Name): The common name for the motif. (Residue and Atom Names): The atom names used to define the motif. Some motifs may match multiple residue types, in which case all matching residues are listed with their corresponding atom names.
(DOCX)

Table S2 **Search parameters for all figures.** (Figure): The figure and sub-figure the selections and searches correspond to. (Selection/{Search}): No braces indicates a saved selection referenced by searches. Braces indicate a search based in terms of previous selections of the form {sel1, sel2, …}. “sc” indicates only the side-chain was taken from the previously saved selection and “bb” indicates only the backbone atoms were used. (Structure): The PDB ID the selection originated from. (Result ID): The search result serial ID number to disambiguate selections where there are multiple results from the same PDB ID. (Chain): Chain the selection originated from. (Residue): Residue selected. (Atoms): Selected atoms. (RMSD Cutoff): Root-mean-squared deviation cutoff used for a given search. With the exception of initial selections for each figure, all selections are derived from results returned from the preceding search query in the table. †: Structure provided by the David Baker laboratory for their hot spot motif for the hemagglutinin binder [20].
(DOCX)

Table S3 **Results for throughput benchmarks.** (Search) The matching column from Figure 8. (Figure #) The figure the search corresponds to. (Search #) The order of searches used to build the structure depicted in the figure (See Table S2). (# of atoms) The number of atoms in the search query. (Time) The elapsed time from query submission to final result retrieval. (Matches) The number of matched results returned by the search engine. (Structures/second) The throughput of the search engine, defined as (Time/Models indexed).
(DOCX)

Manual S1 **User instructions.** These instructions provide detailed guidance on how to use all three software packages (Software S1, S2, S3).
(PDF)

Software S1 **PyMOL search plugin.** This software package contains the source code for building the search plugin, released under a BSD license. This package builds both a plugin suited for PyMOL's plugin manager as well as an alternative Debian package for installation on systems using Debian-like package managers (such as Ubuntu).
(ZIP)

Software S2 **Command line search client.** This software package contains the source code for the command line search client, released under a BSD license. This package also contains the original search queries and results used for this manuscript (Table S2) saved as PyMOL sessions. The test suite for this package uses the command line client to automate these searches and verify that they match the original output we obtained. The benchmark suite for this package automates the benchmarks provided in this paper. The README.md file contains instructions for how to install and test the software.
(ZIP)

Software S3 **Search engine.** This software package contains the source code for the search engine, released under a GPLv2 license. Installation instructions are contained within the README.md file.
(ZIP)

Video S1 **Example use of the PyMOL Suns plugin.** The search process mirrors Figure 3, with the exception of the final search, which is performed at an RMSD cutoff of 0.4 Å. The process begins by selecting a guanidium group and performing a search to visualize neighboring motifs, followed by selection of a nearby carboxylic acid (see: Figure 3A). Repeating the search reveals several matching salt bridges. Search results are reoriented to show the distribution of linker preferences for arginine and one such linker is selected (see: Figure 3B). The final search includes the linker and the matching results are reoriented to center on several backbone motifs that complete the salt bridge. The video has not been sped up, cut, or edited in any way.
(MP4)
