# Supplementary material for: A Real-Time All-Atom Structural Search Engine for Proteins
Source: PLoS Comput Biol. 2014 Jul 31;10(7):e1003750. doi: 10.1371/journal.pcbi.1003750 (PMC4117414; doi:10.1371/journal.pcbi.1003750)
Supplement: Table S1 — Default motif set. Default motifs indexed by the public server hosted at suns.degradolab.org. (Motif Name): The common name for the motif. (Residue and Atom Names): The atom names used to define the motif. Some motifs may match multiple residue types, in which case all matching residues are listed with their corresponding atom names. (DOCX) [file pcbi.1003750.s001.docx]

| Motif Name | Residue and Atom Names |
| --- | --- |
| Alanine | Ala(Cα,Cβ) |
| Arginine Linker | Arg(Cα,Cβ,Cγ,Cδ) |
| Asparagine Linker | Asn(Cα,Cβ,Cγ) |
| Aspartate Linker | Asp(Cα,Cβ,Cγ) |
| Carboxamide | Asn(Cγ,Oδ,Nδ), Gln(Cδ,Oε,Nε) |
| Carboxyl | Asp(Cγ,Oδ1,Oδ2), Glu(Cδ,Oε1,Oε2) |
| Cysteine | Cys(Cα,Cβ,Sγ) |
| Glutamine Linker | Gln(Cα,Cβ,Cγ,Cδ) |
| Glutamate Linker | Glu(Cα,Cβ,Cγ,Cδ) |
| Guanidinium | Arg(Cδ,Nε,Cζ,Nη1,Nη2) |
| Histidine Linker | His(Cα,Cβ,Cγ) |
| Hydroxyl | Ser(Cβ,Oγ), Thr(Cβ,Oγ), Tyr(Cζ,Oη) |
| Imidazole | His(Cγ,Cδ,Nδ,Cε,Nε) |
| Indole | Trp(Cγ,Cδ1,Cδ2,Cε1,Cε2,Nε,Cζ1,Cζ2,Cη) |
| Isoleucine | Ile(Cα,Cβ,Cγ1,Cγ2,δ) |
| Lysine End | Lys(Cδ,Cε,Nζ) |
| Lysine Linker | Lys(Cα,Cβ,Cγ,Cδ) |
| Methionine End | Met(Cγ,Sδ,Cε) |
| Methionine Linker | Met(Cα,Cβ,Cγ) |
| Peptide Bond | All Residues(Cα,C,N,O) |
| Phenylalanine Linker | Phe(Cα,Cβ,Cγ) |
| Phenyl | Phe(Cγ,Cδ1,Cδ2,Cε1,Cε2,Cζ), Tyr(Cγ,Cδ1,Cδ2,Cε1,Cε2,Cζ) |
| Proline Ring | Pro(Cβ,Cγ,Cδ) |
| Serine Linker | Ser(Cα,Cβ) |
| Threonine Linker | Thr(Cα,Cβ,Cγ) |
| Tryptophan Linker | Trp(Cα,Cβ,Cγ) |
| Tyrosine Linker | Tyr(Cα,Cβ,Cγ) |
| Valine | Val(Cα,Cβ,Cγ1,Cγ2) |
