# Supplementary material for: A Real-Time All-Atom Structural Search Engine for Proteins
Source: PLoS Comput Biol. 2014 Jul 31;10(7):e1003750. doi: 10.1371/journal.pcbi.1003750 (PMC4117414; doi:10.1371/journal.pcbi.1003750)
Supplement: Table S2 — Search parameters for all figures. (Figure): The figure and sub-figure the selections and searches correspond to. (Selection/{Search}): No braces indicates a saved selection referenced by searches. Braces indicate a search based in terms of previous selections of the form {sel1, sel2, …}. “sc” indicates only the side-chain was taken from the previously saved selection and “bb” indicates only the backbone atoms were used. (Structure): The PDB ID the selection originated from. (Result ID): The search result serial ID number to disambiguate selections where there are multiple results from the same PDB ID. (Chain): Chain the selection originated from. (Residue): Residue selected. (Atoms): Selected atoms. (RMSD Cutoff): Root-mean-squared deviation cutoff used for a given search. With the exception of initial selections for each figure, all selections are derived from results returned from the preceding search query in the table. †: Structure provided by the David Baker laboratory for their hot spot motif for the hemagglutinin binder [20]. (DOCX) [file pcbi.1003750.s002.docx]

| Figure | Selection / {Search} | Structure | Result ID | Chain | Residue | Atoms | RMSD Cutoff (Å) |
| --- | --- | --- | --- | --- | --- | --- | --- |
| 2 | 1 | 2GBP | N/A | A | Arg4 | Cδ,Nε,Cζ,Nη1,Nη2 |  |
|  | {1} |  |  |  |  |  | 0.2 |
|  | 2 | 3A6R | 1 | A | Asp61 | Cγ,Oδ1,Oδ2 |  |
|  | {1,2} |  |  |  |  |  | 0.2 |
|  | 3 | 3P02 | 0 | A | Arg325 | Cα,Cβ,Cγ,Cδ |  |
|  | {1,2,3} |  |  |  |  |  | 0.3 |
| 3A | 4 | 2GBP | N/A | A | Val88 | Entire Residue |  |
|  | {4} |  |  |  |  |  | 0.1 |
|  | 5 | 4ASM | 0 | B | Val353 | Entire Residue |  |
|  | 6 | 2WUR | 0 | A | Tyr92 | Entire Residue |  |
| 3B | {4bb,6bb} |  |  |  |  |  | 0.2 |
|  | 7 | 2JCQ | 1 | A | Thr151 | Entire Residue |  |
|  | {4bb,7} |  |  |  |  |  | 0.2 |
|  | 8 | 2JCQ | 0 | A | Thr149 | Entire Residue |  |
|  | {7sc,8bb} |  |  |  |  |  | 0.5 |
|  | 9 | 3B34 | 0 | A | Thr37 | Entire Residue |  |
|  | {5bb,8sc} |  |  |  |  |  | 0.5 |
|  | 10 | 3SUU | 0 | A | Asp102 | Entire Residue |  |
|  | {6bb,7sc} |  |  |  |  |  | 0.5 |
|  | 11 | 3D9A | 0 | H | Thr482 | Entire Residue |  |
|  | {6bb,8sc} |  |  |  |  |  | 0.5 |
|  | 12 | 3Q1I | 0 | A | Thr561 | Entire Residue |  |
| 4A | 13 | † | N/A | B | Met503 | Cγ,Sδ,Cε |  |
|  | 14 | † | N/A | B | Phe504 | Cγ,Cδ1,Cδ2,Cε1,Cε2,Cζ |  |
|  | {13,14} |  |  |  |  |  | 0.7 |
| 4B | {7sc,8sc} |  |  |  |  |  | 0.6 |
