# Supplementary material for: A Real-Time All-Atom Structural Search Engine for Proteins
Source: PLoS Comput Biol. 2014 Jul 31;10(7):e1003750. doi: 10.1371/journal.pcbi.1003750 (PMC4117414; doi:10.1371/journal.pcbi.1003750)
Supplement: Manual S1 — User instructions. These instructions provide detailed guidance on how to use all three software packages (Software S1, S2, S3). (PDF) [file pcbi.1003750.s004.pdf]

Suns: The Structural Search Engine

Suns provides the missing search engine for the Protein Data Bank complete with a PyMOL interface:

- Point and click within PyMOL to build search queries
- Search for sophisticated all-atom motifs
- PyMOL streams aligned search results into your session
- Interactively build motifs using search results
- Validate modeled or designed structures against real crystal structures

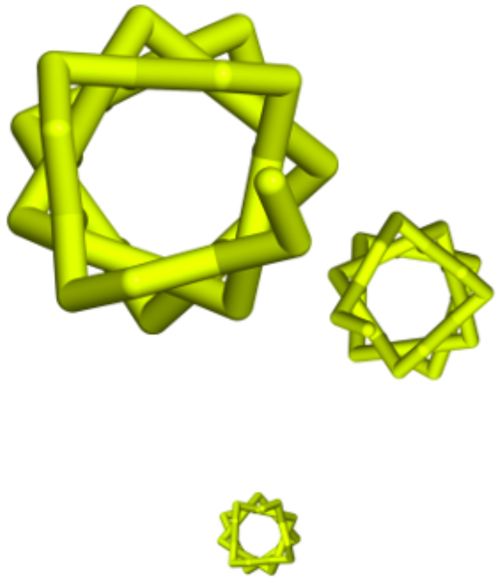

PyMOL Plugin

The easiest way to use Suns is through the PyMOL plugin. You can install this plugin in two ways:

- Using PyMOL's plugin manager (only available to latest incentive builds of PyMOL).
- Using a Debian/Ubuntu package (assuming you also installed PyMOL this way, too)

Requirements

- PyMOL
- An internet connection (or a locally installed Suns search engine)

Install using Plugin Manager

Download Plugin

- Download the Suns plugin (suns.zip).

- Open `PyMOL`
- Select `Plugin -> Plugin Manger` from the menu
- Go to the `Install New Plugin` tab
- Click `Choose file...` and select the downloaded plugin (`suns.zip`)

Install using Debian/Ubuntu Package

Download Package

- Download the `pymol-suns-search` package
- Install the package using your system's package manager

Tutorial

Start `PyMOL` and load the structure for glucose binding protein (PDB ID: `2GBP`). You can either:

- use the `Plugin -> PDB Loader Service` menu option and choose `2gbp`, or
- type `fetch 2gbp` at the `PyMOL` command prompt.

- 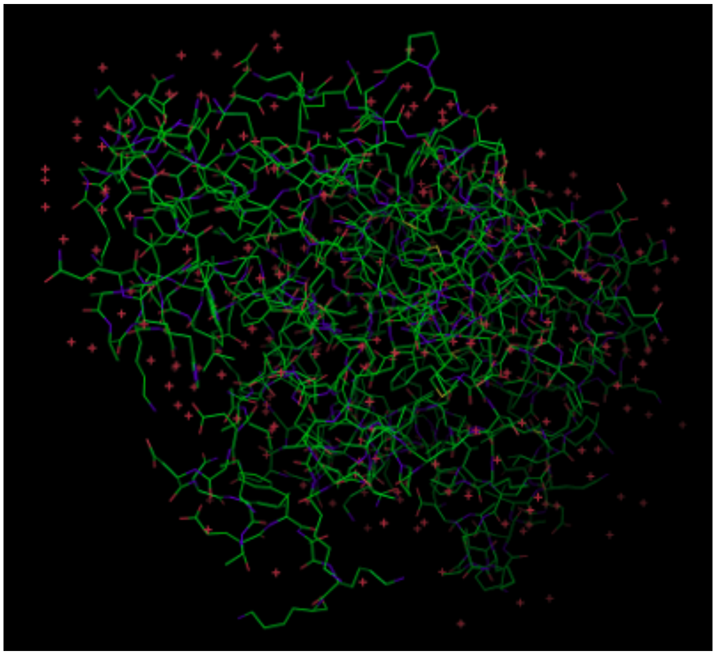

  Glucose binding protein - PDB ID `2gbp`

Select the `Plugin -> Suns Search` menu option to open the search wizard, which lets you build and submit structural search queries:

- 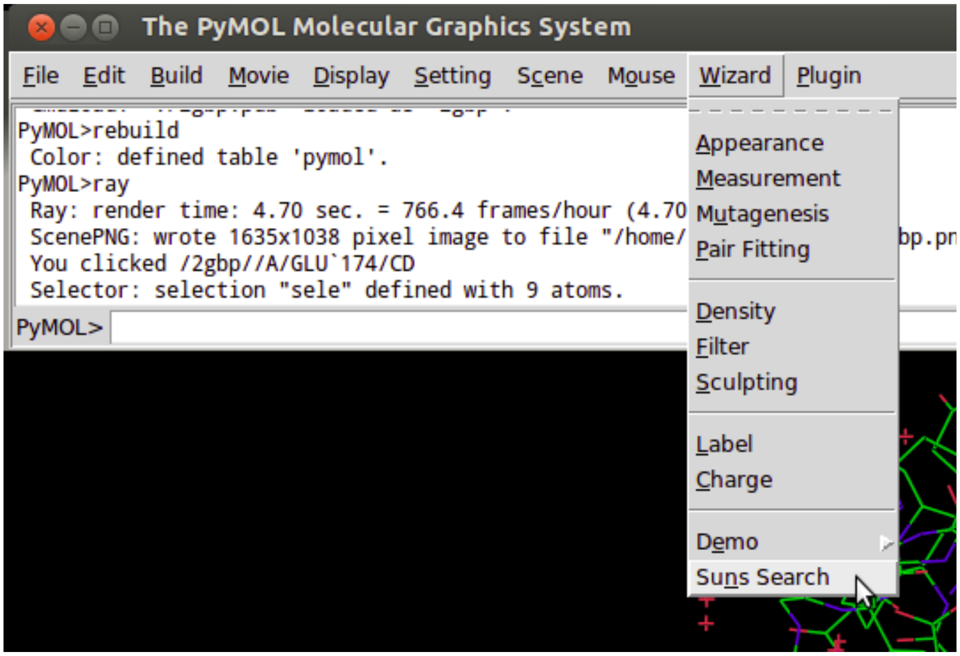

  The wizard menu

- 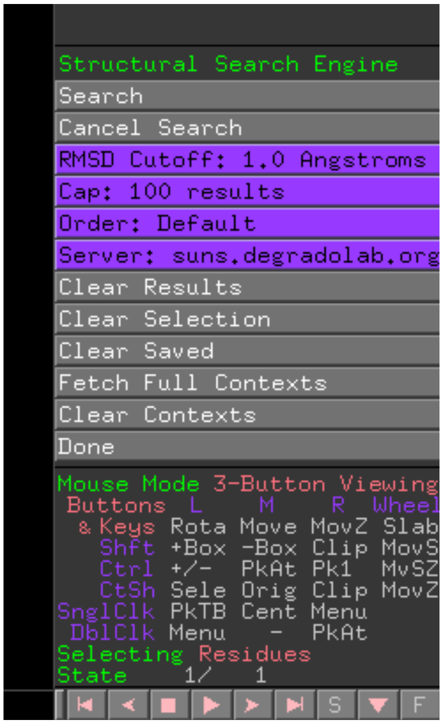

  The search wizard

Left-click a carboxyl group to select it and add it to your search query:

- 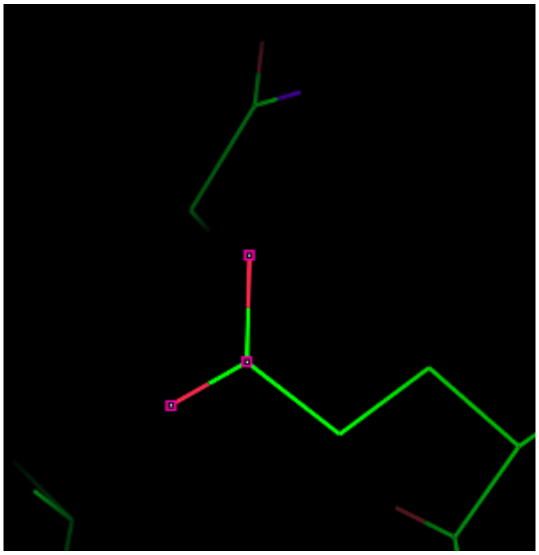

  Selected carboxyl group

`Suns` pre-indexes chemical motifs from protein structures and the `PyMOL` search wizard automatically expands your selection to pre-indexed motifs, including:

- carboxyls,
- peptides,
- hydroxyls, and
- phenyl groups.

Click "Search" to search for the carboxyl motif. The search wizard contacts the public search engine at `suns.degradolab.org` and streams in aligned search results:

- 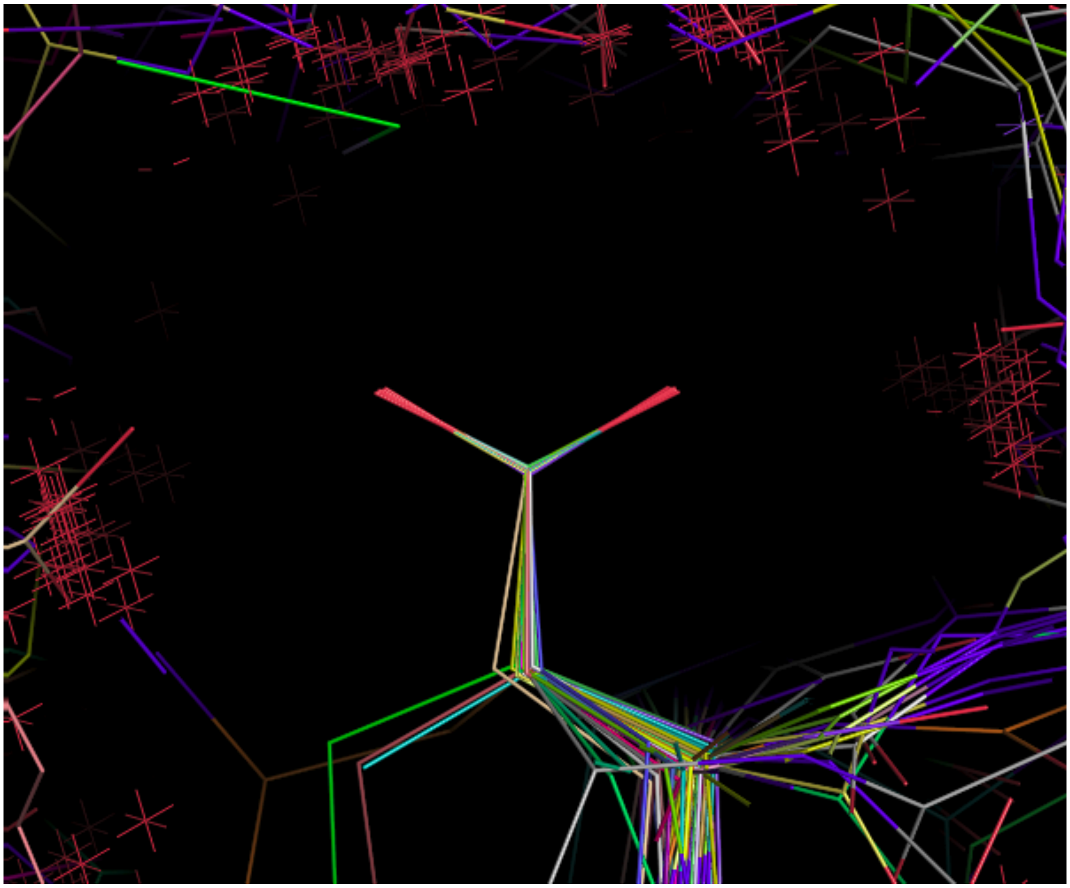

  Carboxyl search results

`PyMOL` uniquely colors each new result, but you can impose a uniform coloring by setting all carbons to green:

- 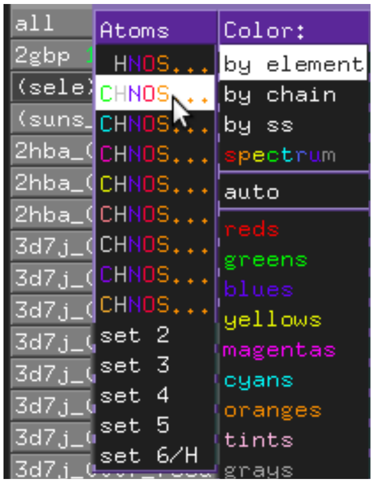

  Set the backbone to green

- 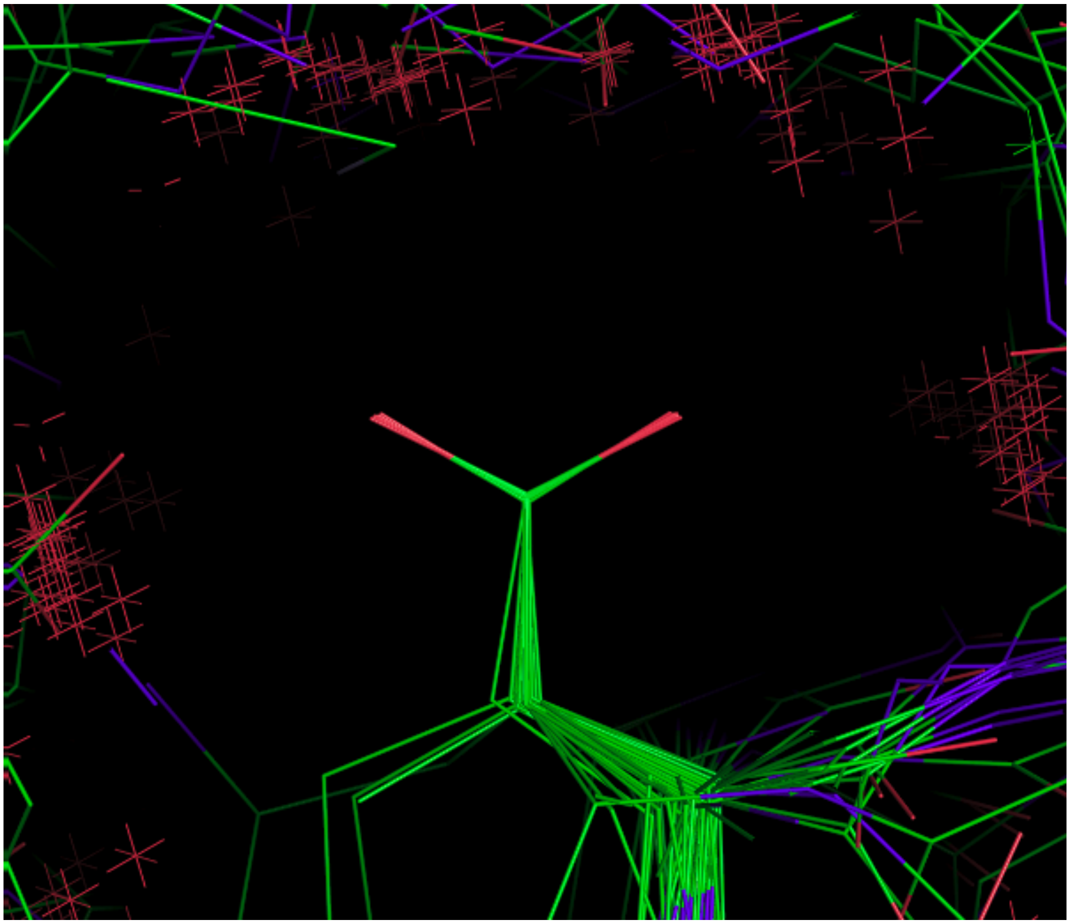

  The re-colored search results

Some motifs match multiple residues. For example, the carboxyl search query will match carboxyls from both aspartate acid and glutamate.

Other motifs uniquely identify residues, such as linkers. Click on any search result's linker and the wizard will expand the selection to include the remainder of the side chain. The linker uniquely identifies the residue as aspartate or glutamate.

- 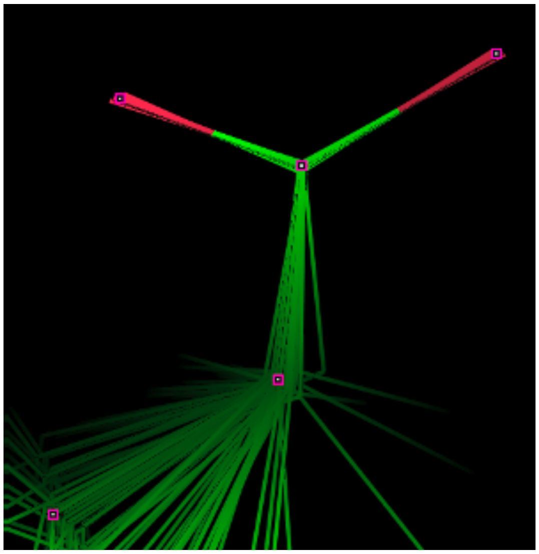

  Selection expanded to include linker

Suns saves search results that you add to your search query by renaming them to end with _save so that future searches don't delete them.

Click "Search" to search for the expanded selection. The new search will automatically delete old search results. To save them, you must copy them to a new object. You can also remove results manually at any time using "Clear Results".

The search engine will return several less-than-perfect matches since searches default to an RMSD cutoff of 1.0 Angstroms:

- 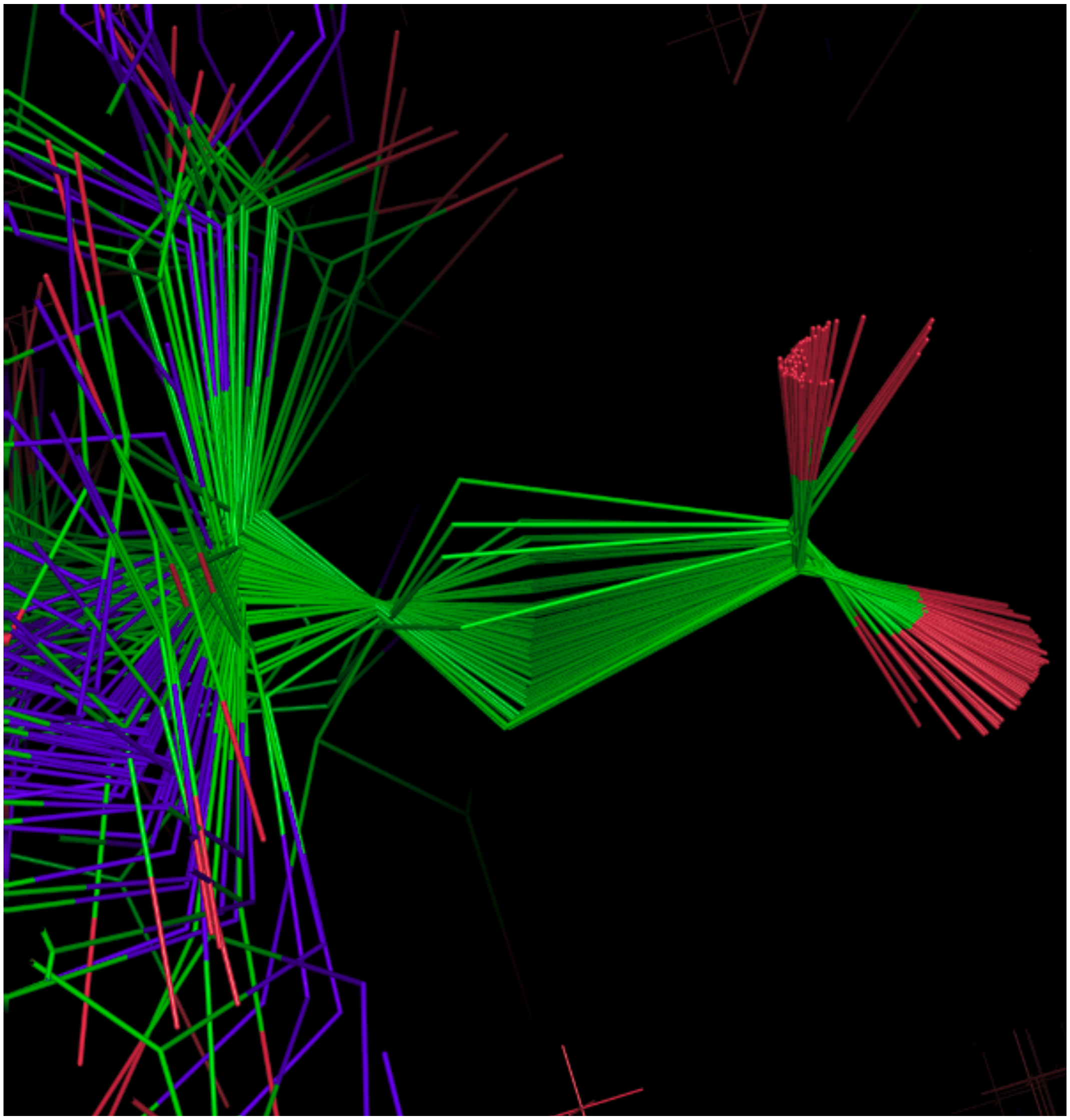

  Loose results

Use the "RMSD Cutoff" option to improve the result quality. Set the RMSD to 0.1 Angstroms and redo the search, which will considerably tighten up the linker:

- 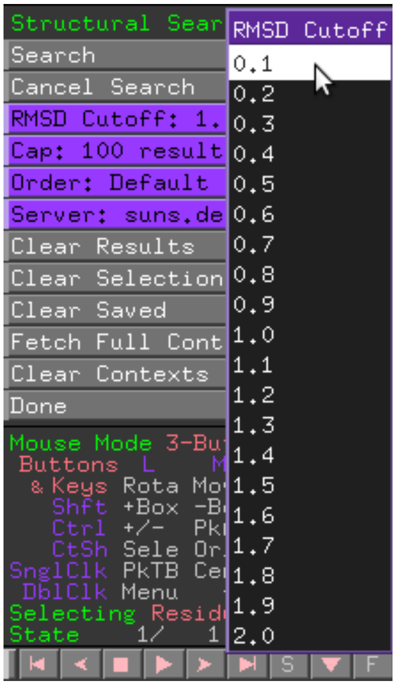

  Change the RMSD Cutoff to 0.1 Angstroms

- 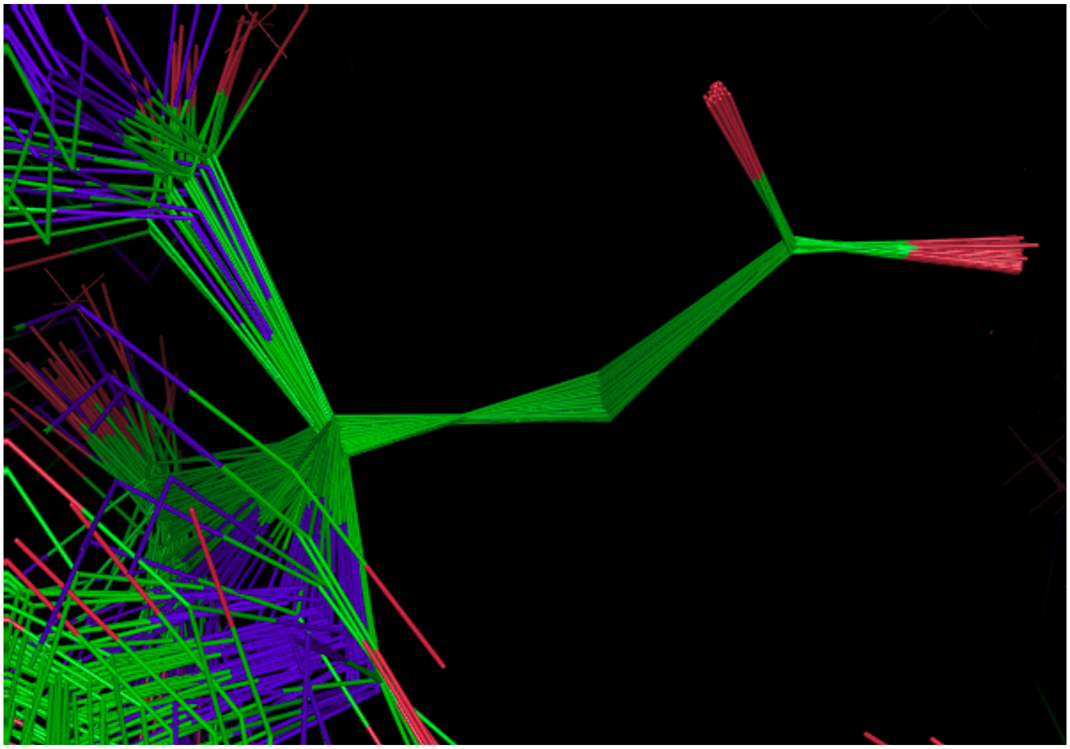

  Tight results

You don't have to limit yourself to contiguous motifs. You can search for disconnected elements, too. Let's hide everything except aspartate and arginine to find a candidate salt bridge:

```
hide everything, not (resn arg or suns_query)
```

This reveals a cluster of arginines:

- 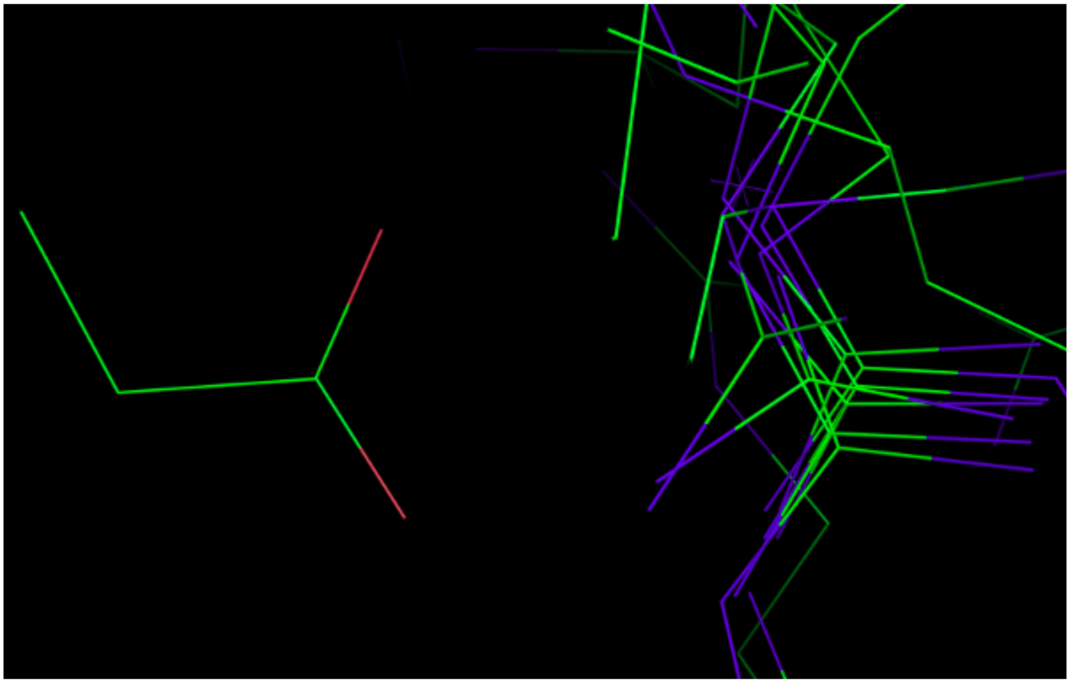

Cluster of arginines near the aspartate

Pick any arginine's guanidinium group and loosen the search cutoff to 0.5 RMSD to search for geometrically similar salt bridges:

- 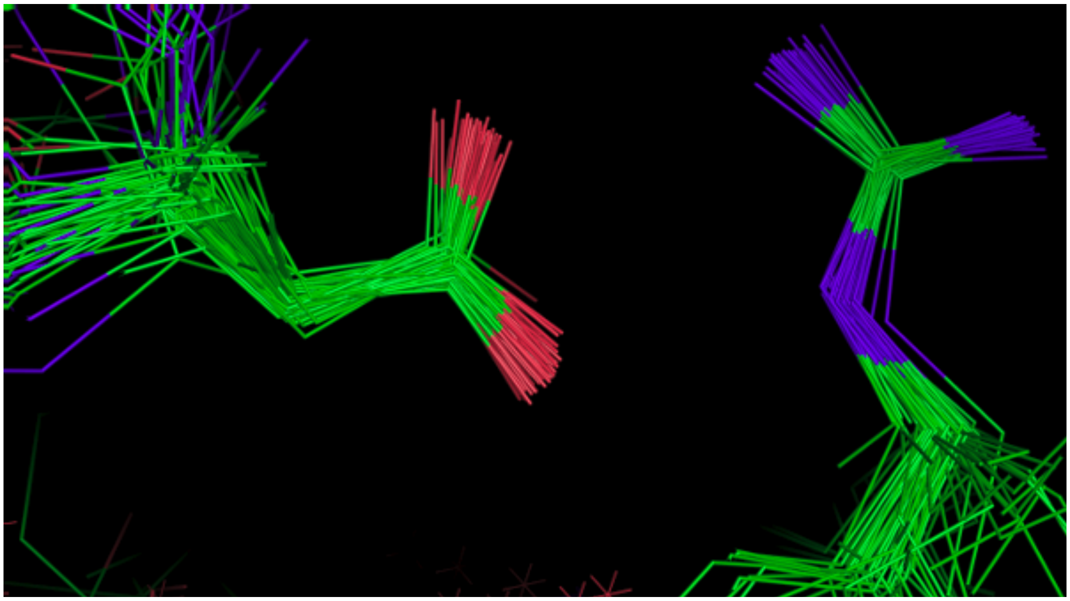

Several identical salt bridge matches

Search results only bring in a 15 Angstrom cube around the match, but sometimes we desire more context for a given search result. To pull in the original structure, just disable all selections except the desired result(s) and click "Fetch Full Contexts":

- 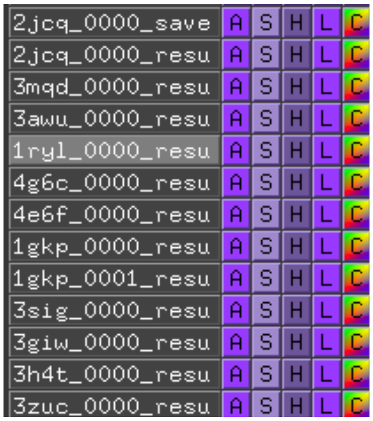

  Enable the selections you wish to expand

- 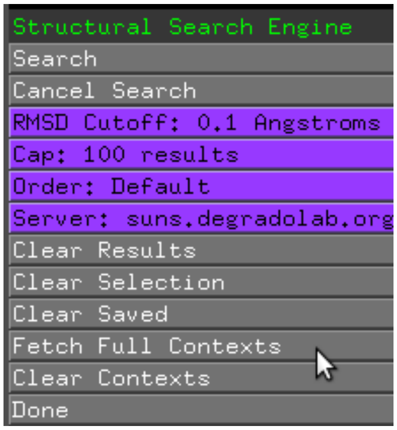

  Click "Fetch Full Contexts"

This will fetch the original structures from the Protein Data Bank and align them to their respective results:

- 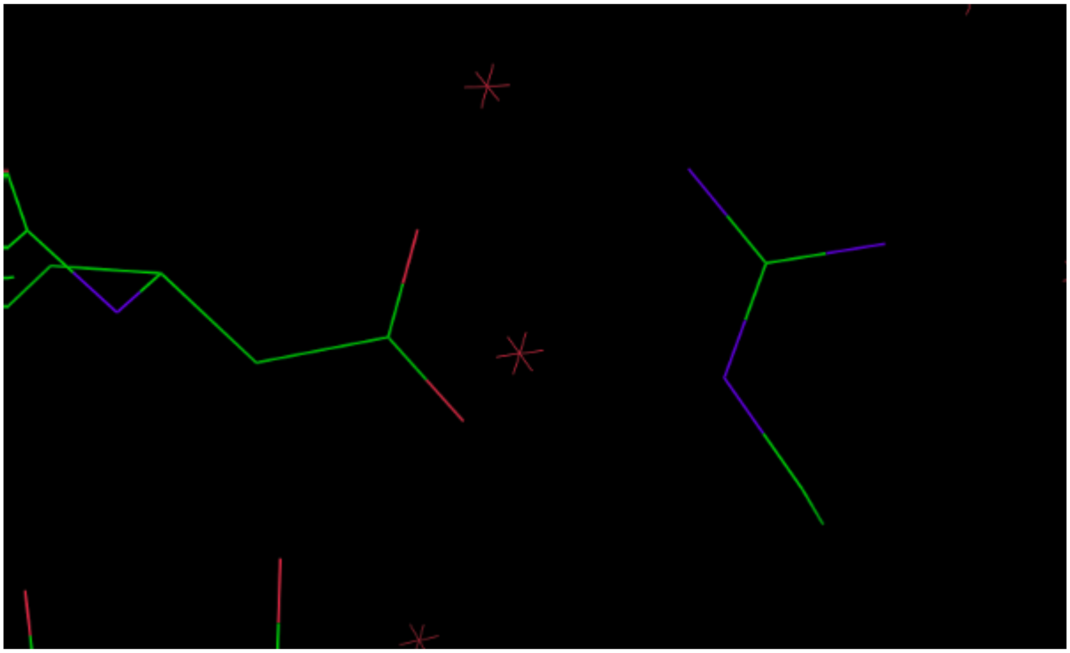

  Before adding context

- 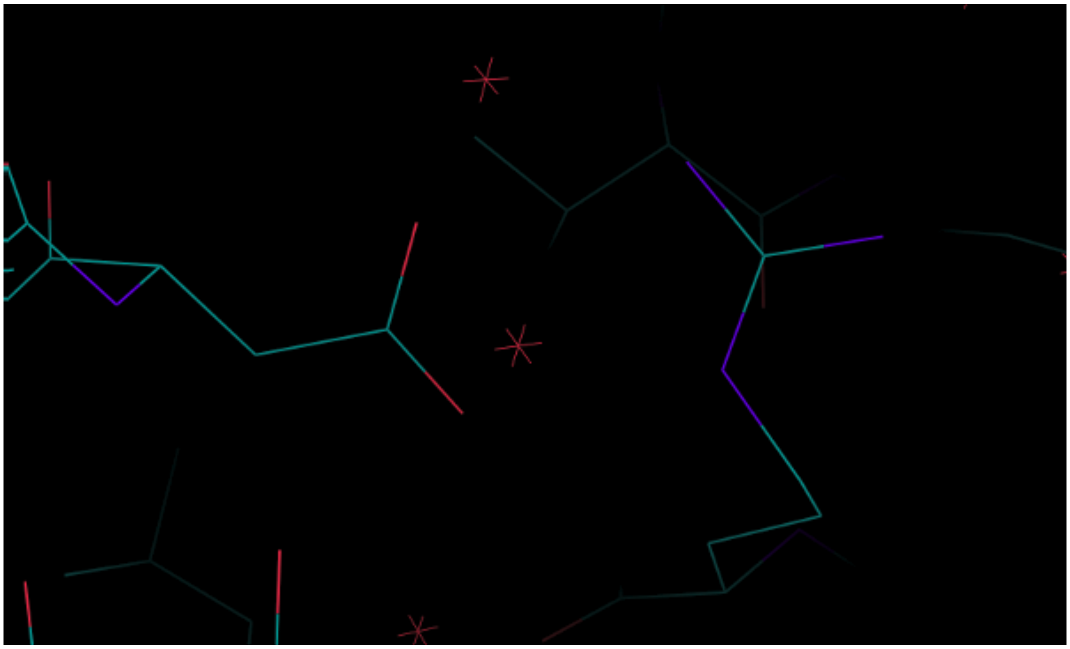

  After adding context

`Suns` also supports backbone peptide searches. Reset the wizard by clicking:

- Clear Results: Removes search results ending with `_result`
- Clear Selection: Empties the `suns_query` selection
- Clear Saved: Removes saved results ending with `_save`
- Clear Contexts: Removes fetched contexts ending with `_fetch`

Then type:

```
show all
```

Now select two consecutive peptide bonds from a helix and search with a cutoff of 0.3 Angstroms. The search results extend the initial query for several helical turns in each direction:

- 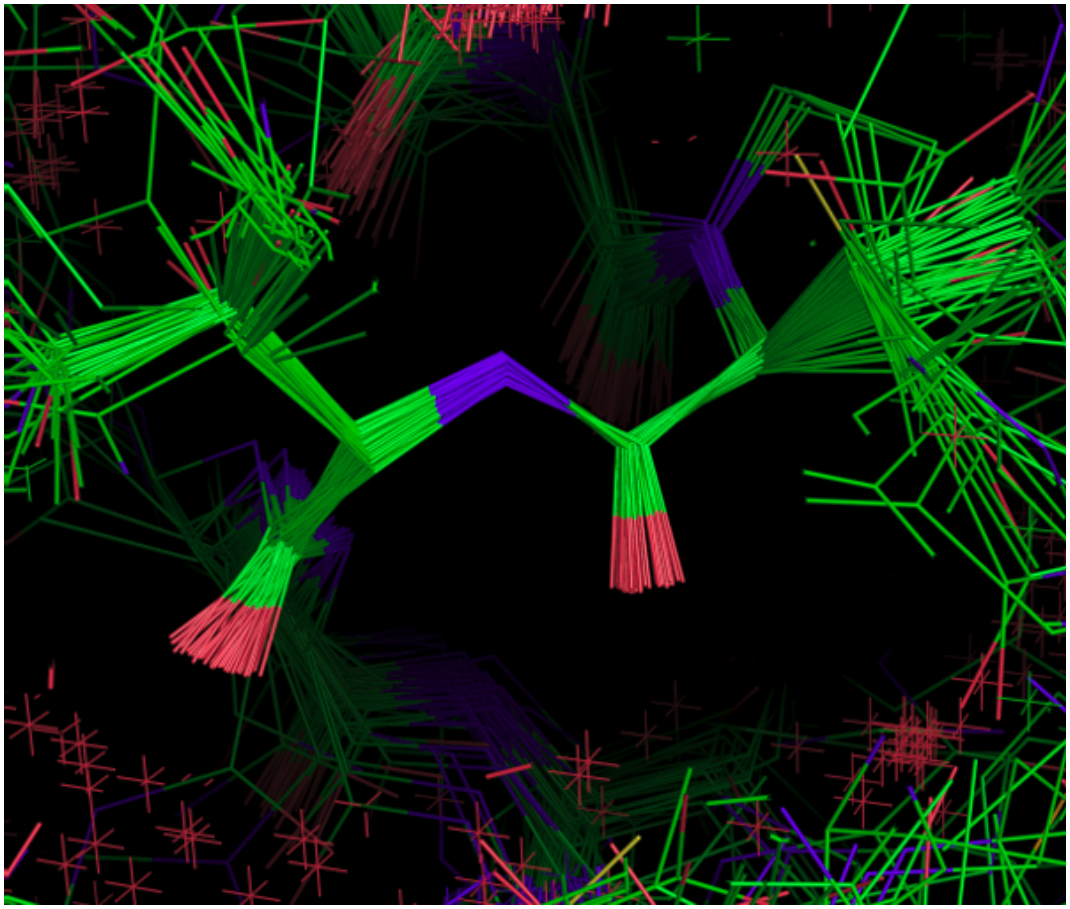

  Inferred helical context

You can also search for residues V207 and V232 and include both the valine and backbone atoms in your search query: in the preferred surrounding backbone:

- 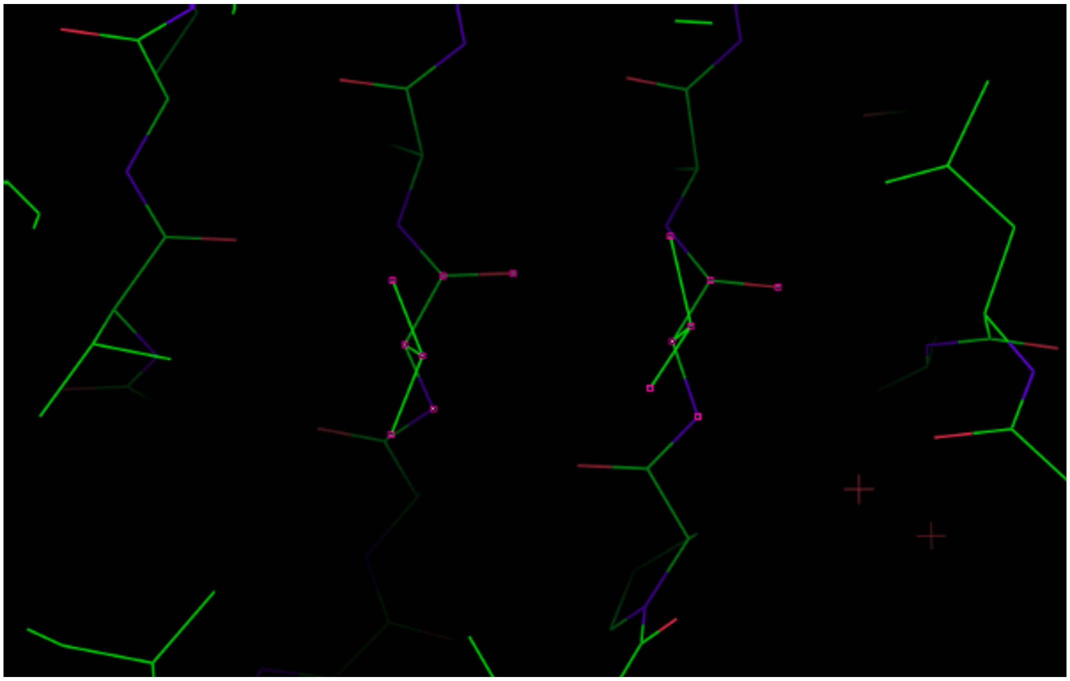

  Double valine selection

Searching at 0.4 Angstrom RMSD cutoff brings in an entire beta sheet worth of context:

- 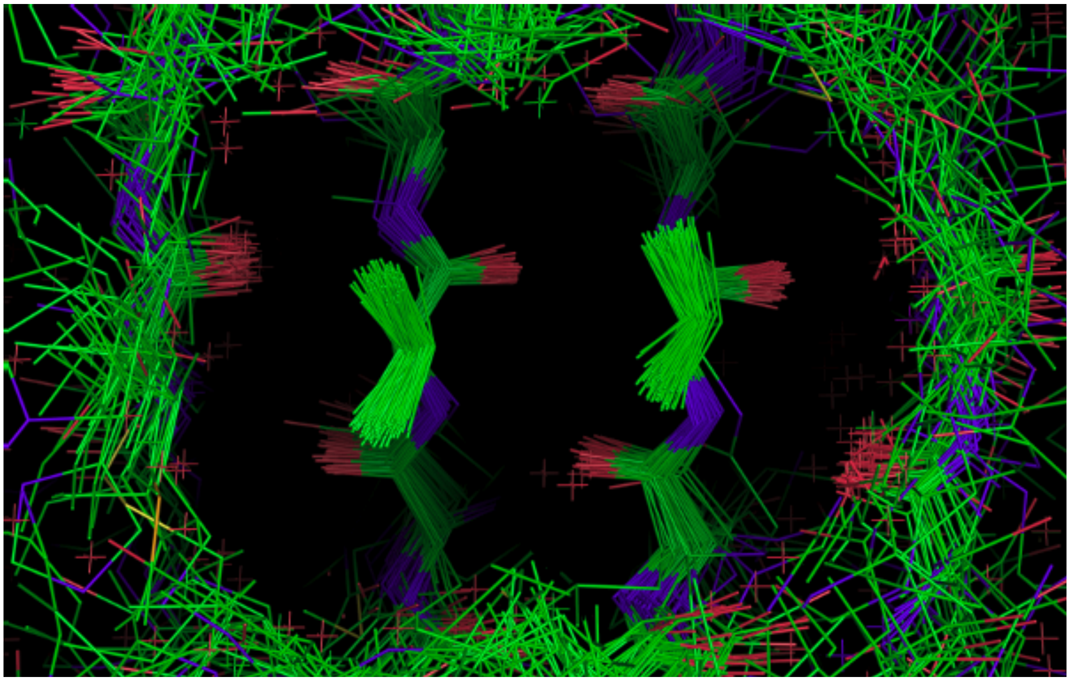

  Inferred beta sheet context

`Suns` plays well with other `PyMOL` wizards, including the mutagenesis wizard. We can combine the two wizards to infer contextual preferences for different rotamers.

Use the mutagenesis wizard to rotate V232 counterclockwise and repeat the search:

- 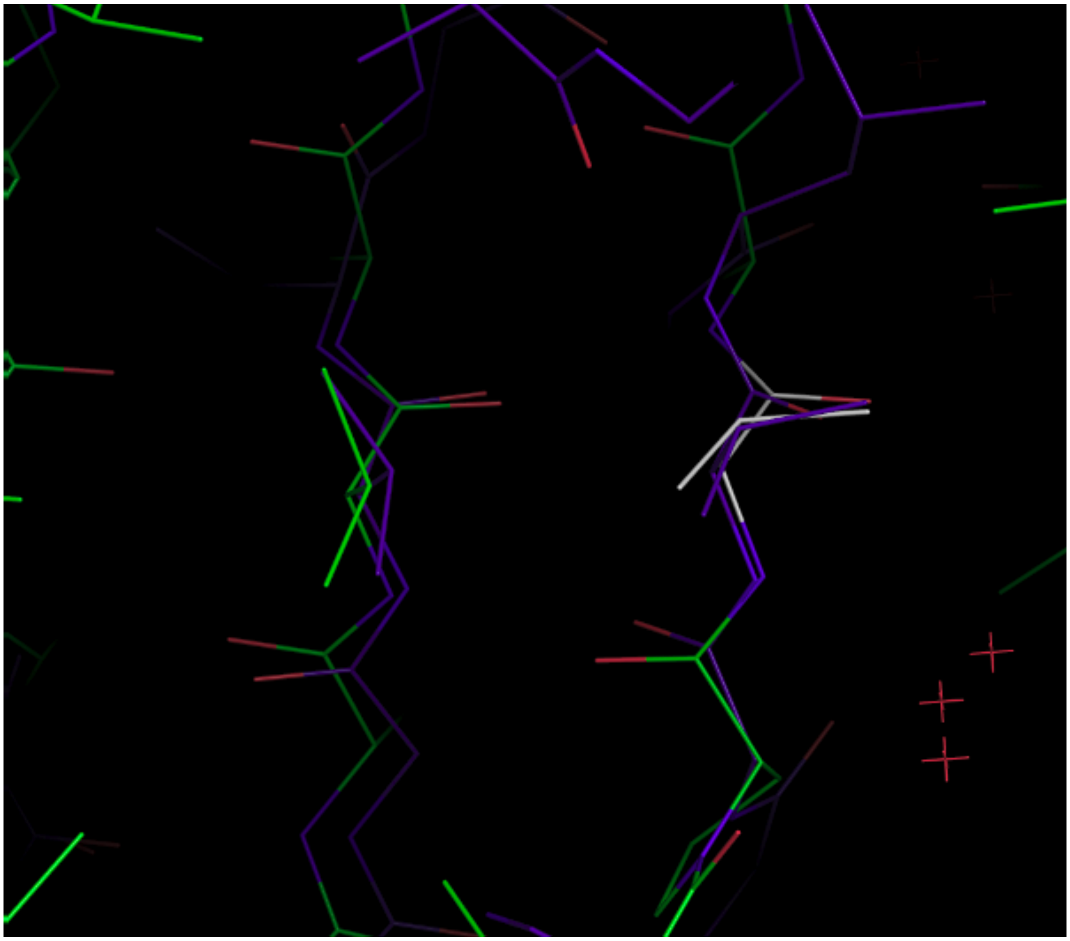

  Only one match to the search query

The number of matches drops precipitously, indicating an unfavorable motif. You can use `Suns` as a crude measure of the quality of modeled or designed protein motifs.

Command Line Client

`suns-cmd` is a command line client to the Suns protein search engine. Use `suns-cmd` to automate searches for scripting purposes.

Installation

You can install the command line client from the following source package:

[Download suns-cmd source]

To install the source package, first install the [Haskell Platform].

```
$ cabal update
$ cabal install
```

Quick Start

To use `suns-cmd`, just create a directory to store the results:

```
$ mkdir results
```

... and feed in the motif to search to the program's standard input. This source package provides example motifs in the `test/` subdirectory:

```
$ ~/.cabal/bin/suns-cmd -d results/ -r 0.2 < test/figure2/search1.pdb
```

The `-d` parameter tells the program to store all results in the `results/` directory:

```
$ ls results
1tqg_0.pdb   1v7w_2.pdb  2fr5_1.pdb  3a6r_0.pdb   3cuz_1.pdb  3fke_0.pdb
1tqg_1.pdb   1v7w_3.pdb  2fr5_2.pdb  3a6r_1.pdb   3cuz_2.pdb  3fke_1.pdb
...
```

Each result is labeled by the structure name followed by a number which distinguishes results originating from the same structure. These results are already aligned to the original search query.

This program also accepts the following options:

```
suns-cmd: The Suns search command line client

Usage: suns-cmd [--hostname STRING] [-r|--rmsd DOUBLE] [-n|--num INT] [-s|--seed INT] [-d|--directory FILEPATH]
  Send search requests and store results as PDB files

Available options:
  -h,--help                Show this help text
  --hostname STRING        Search engine address (default: suns.degradolab.org)
  -r,--rmsd DOUBLE         RMSD cutoff (default: 1.0)
  -n,--num INT             Number of results (default: 100)
  -s,--seed INT            Randomization seed (default: 0)
  -d,--directory FILEPATH  Results directory (default: ./)

Report bugs to suns-search@googlegroups.com
```

Search Engine

The public `Suns` server at `suns.degradolab.org` imposes three limitations in order to handle a high search volume:

- Queries must fit inside a 15 Angstrom box
- You can only search on pre-defined protein substructures
- Searches time out after 10 seconds

If you set up your own local search engine, you can bypass these limitations, allowing you to:

- Change the database of indexed protein structures
- Index ligands or alternative protein substructures
- Run search queries without time limits

For instructions on how to do this, download the source package at https://github.com/Gabriel439

/suns-cmd and follow the instructions to build and customize your own local search engine.

Support

To report bugs, request features, or ask for support, contact the official mailing list at
`suns-search@googlegroups.com`.
