# Supplementary material for: A Real-Time All-Atom Structural Search Engine for Proteins
Source: PLoS Comput Biol. 2014 Jul 31;10(7):e1003750. doi: 10.1371/journal.pcbi.1003750 (PMC4117414; doi:10.1371/journal.pcbi.1003750)
Supplement: Supplementary file 4 [file pcbi.1003750.s003.docx]

Search	Figure #	Search #	# of atoms	Time (ms)	Matches	Structures / second
Suns - 1	3	1	4	17474	11856	120.8081
Suns - 2	3	2	8	1357	114	1555.637
Suns - 3	3	3	12	1556	43	1356.684
Suns - 4	4	1	7	4222	601	500
Suns - 5	4	2	8	235740	3768	8.954781
Suns - 6	4	3	11	12952	521	162.9864
Suns - 7	4	4	8	12890	375	163.7704
Suns - 8	4	5	8	12718	482	165.9852
Suns - 9	4	6	8	12758	444	165.4648
Suns -10	4	7	8	13398	756	157.5608
Suns - 11	5B	8	8	880	82	2398.864
Suns -12	5A	1	9	419	31	5038.186
Erebus - 1	N/A	N/A	7	9000000	6362	22.22222
Erebus - 2	N/A	N/A	10	6780000	4415	29.49853
Erebus - 3	N/A	N/A	13	5760000	4	34.72222
Erebus - 4	N/A	N/A	13	6240000	6	32.05128
Erebus - 5	N/A	N/A	13	6420000	7	31.15265
Erebus - 6	N/A	N/A	16	6000000	1	33.33333
Erebus - 7	N/A	N/A	16	7320000	251	27.3224
Erebus - 8	N/A	N/A	25	2E+07	22516	9.920635

Models inc	Models indexed (Erebus)
